# DETERMINANTS OF DEPRESSION AMONG AGING LGBT IN TEXAS

**DOI:** 10.1093/geroni/igz038.994

**Published:** 2019-11-08

**Authors:** Ami Moore

**Affiliations:** University of North Texas, Denton, Texas, United States

## Abstract

Introduction: Aging LGBT experience significant health disparities compared to their heterosexual counterparts. As a sexual minority, they have a high likelihood of acquiring physical and mental illnesses. Methods: This study investigated factors that are associated with reports of depression among LGBT. Data came from Project Gray Pride, a study of aging gay, lesbian, bisexual, and transgender men and women living in a metropolitan area in Texas (N=126). Linear regression analysis was used. Results: Results show a negative association between respondents who self-identified as cisgender female and depression compared to those who self-identified as transgender. However, social vigilance and reports of health symptoms positively correlate with depression. Additionally, the interaction term of gender and race showed that respondents who were non-Whites and female reported higher levels of depression relative to their counterparts who were White males or White transgender. Other factors such as HIV status, age, education were not statistically significant. Conclusion: This study shows that being socially vigilant increases depression among aging LGBT. Also, aging female LGBT who are non-White may have an increased burden of negative social experiences leading to high levels of depression. As the population of older LGBT is increasing, intervention efforts must take into account these important findings in order to devise sound policies and programs to improve mental health among LGBT.

